# Providing Safe and Effective Surgical Care During the COVID-19 Outbreak in the UK – Changing Strategies

**DOI:** 10.34172/ijhpm.2020.112

**Published:** 2020-06-30

**Authors:** Robert Chapman, S Guru Naidu, Manoj Nair, Laura Spanu, Jasdeep Gahir

**Affiliations:** Department of General Surgery, North Middlesex University Hospital, London, UK.

## Dear Editor,

Healthcare systems were stretched to the limit by the coronavirus disease 2019 (COVID-19) outbreak. The increase in demand for staff, space and resources to treat patients with COVID-19 meant that patients requiring other forms of emergency care experienced improvised systems and pathways. The impact of these pressures particularly affected the provision of acute surgical care within the United Kingdom. The central response included the cancellation of all non-urgent elective surgery and the sequestration of operating theatre space as critical care overspill areas.^1^ However, local responses were dependent on initial surgical resources and local COVID-19 burden. In this letter, we describe measures implemented by the general surgical department at a university teaching hospital in North London to ensure adequate provision of emergency surgical care.

### Access to Surgical Services

Acute surgical admissions are usually assessed by the on-call surgical registrar in the emergency department (ED), and then accepted for admission or discharged. However, during the COVID-19 outbreak this system was not workable due to the risk of exposing surgical patients and staff to COVID-19 in ED, and secondly a need to try and ambulate as many patients as possible in an attempt to reduce admissions and protect inpatient resources for COVID-19 patients.

The surgical team developed a new patient pathway to maintain quality of care, as well as patient and staff safety. The pathway began with a senior surgical nurse specialist identifying patients that warrant further surgical assessment and moving them straight to an Emergency Ambulatory Surgical Unit (EASU). The criteria for identifying these patients were based on presenting complaint, radiological and biochemical findings, as well as discussion with the ED doctors. Notably, all patients triaged to EASU had a COVID-19 swab test prior to leaving the ED, and all members of staff in EASU wore full PPE protection to ensure the safety of the surgical workforce, and to protect other patients from cross-infection. Further safety precautions included the requirement of cross-sectional chest imaging prior to any surgical procedure to ensure the patient did not have a COVID-19 pneumonia.

Once at EASU, the on-call surgical team would further assess the patient and make a decision on how to proceed. The objective was to admit only those patients that truly needed inpatient treatment or emergency surgery. The remaining patients were either discharged without follow-up or asked to return to EASU to ensure clinical improvement (Figure). The decision to admit or discharge was largely dependent on whether there was a need to operate imminently (within 12 hours of assessment). If the patient did not need emergency surgery or acute medical care then the patient would be discharged and monitored in the community or via follow-up at EASU. Importantly, EASU provides easy and fast access to various investigations, for example a patient may be sent home and then asked to return the following day for an ultrasound scan or a magnetic resonance cholangiopancreatography. The ability to ambulate patients in this way reduces inpatient stays whilst maintaining direct access to inpatient investigations. This method of assessment, with a clear pathway to admission or discharge meant that we were able to access our most unwell patients and efficiently discharge our stable patients, whilst maintaining patient safety and minimizing our use of inpatient hospital resources.

**Figure:**
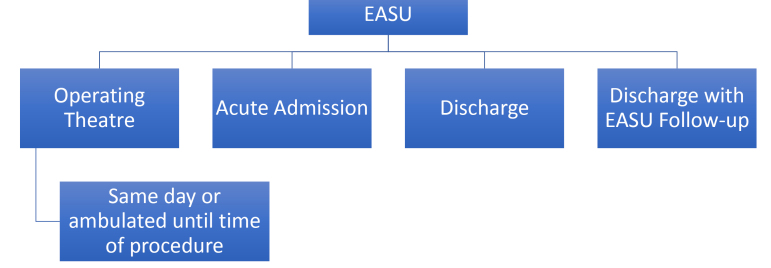
New Clinical Pathway for Emergency Surgical Patients During the COVID-19 Outbreak. The pathway begins when patients are triaged to EASU, and from there they may proceed to one of four clinical outcomes. The patient may be acutely unwell and require immediate surgical intervention (far left box, Operating Theatre). Conversely, the patient may be deemed safe for discharge from surgical services (box second from the right) and be able to safely return home within hours. The other two options are for patients who require an acute admission for hospital-level care (box second from left) or those who can be discharged to the community, but require careful and early follow-up for further investigation or to ensure clinical improvement (far right box). Additionally, there is a stratification of those patients that need surgery, as previously mentioned some may be sent straight to theatre from EASU within hours. However, other patients may be ambulated from EASU and then asked to return to theatre the following day, this option is for patients that require emergency surgery, but their operation is not as time-sensitive and can therefore be postponed for 24 hours or more and further planned. Overall, the pathway has an increased emphasis on the ambulation of patients and outpatient management in an effort to greatly reduce surgical inpatient numbers. Abbreviations: COVID-19, coronavirus disease 2019; EASU, Emergency Ambulatory Surgical Unit.

### Staffing

Normal levels of staffing within our surgical department include twelve consultants, twelve registrars, four senior house officers and twelve foundation doctors. The COVID-19 outbreak saw the transfer of all of our foundation doctors and senior house officers to an emergency COVID-19 rota. In addition, various consultants and registrars were required to self-isolate throughout the outbreak, while others were required to operate on cancer patients at separate hub hospitals,^2^ meaning that at any one time, the department was operating with roughly 50% (or less) of normal staffing levels. More specifically, at times throughout the pandemic, almost 33% of registrars and consultants were self-isolating, putting an immense strain on the remaining members of senior staff.

This issue was overcome by the design and use of an emergency surgical rota that increased registrar and consultant workload to cover the loss of other staff. New roles were introduced at short notice to cover busy periods, such as a twilight registrar. A designated ambulatory care registrar was also introduced to ensure our high-risk patients, who may have previously been admitted, were cared for safely whilst remaining in the community. The rota also allocated ‘back-up’ members of staff who were on stand-by in case colleagues were ill or required to self-isolate at short notice. Furthermore, members of staff who were shielding from COVID-19 were also fulfilling roles by running telephone clinics. The rota was reviewed weekly to ensure staff numbers were meeting the demands of inpatient workload, emergency operating lists and assessing acute admissions. Rota reviews also allowed changes to be made in relation to the current number of staff available to work. For example, when staff had completed self-isolation and were safe to work again, the rota coordinator was informed and the rota re-mapped to reflect available staffing. The flexibility of the department was essential to the continued delivery of safe surgical care throughout the pandemic.

## Conclusion

The COVID-19 outbreak put a unique and unexpected stress on surgical resources across the country. The central response rightly focused on ensuring our healthcare system had capacity for COVID-19 patients. However, the re-organization of other specialties impacted by these changes was very much a local response. Emergency surgical departments had to act rapidly to ensure a safe and effective service could be provided with greatly reduced staffing, inpatient capacity and operating theatre capacity. This report shows how a new patient flow pathway was used to mitigate the dangers of COVID-19 by reducing patient contact and by shifting the focus away from inpatient assessment to ambulatory care. Additionally, the clinician-led re-design and constant review of surgical staffing meant that staff could work safely and effectively throughout the evolving outbreak. These conclusions should be taken into account when designing new systems for surgical patient care and staff provision as we recover from COVID-19 and in preparation for any potential future outbreaks of COVID-19.

## Ethical issues

Not applicable.

## Competing interests

Authors declare that they have no competing interests.

## Authors’ contributions

RC wrote the letter, after the idea was conceived by SGN. The remaining authors reviewed and edited the letter, and were also instrumental in making the pathway and rota changes.
